# The Etiopathogenesis of Preeclampsia: Where Do We Stand Now?

**DOI:** 10.3390/jcm14227992

**Published:** 2025-11-11

**Authors:** Marzena Laskowska, Anna Bednarek, Maciej Stworowski

**Affiliations:** 1 Department of Obstetrics and Perinatology, Faculty of Medicine, Medical University of Lublin, K. Jaczewskiego 8 Street, 20-095 Lublin, Poland; 2 Department of Health Promotion, Nursing Development, Faculty of Health Sciences, Medical University of Lublin, S. Staszica 4 Street, 20-081 Lublin, Poland; 3 Medical Faculty, Medical University of Lublin, Al. Racławickie 1 Street, 20-059 Lublin, Poland

**Keywords:** preeclampsia, etiopathogenesis, fetal growth restriction (FGR), pregnancy complication, placenta

## Abstract

Preeclampsia is a multisystem disorder that develops during pregnancy and is associated with severe complications for both the pregnant woman and her infant. It remains a leading cause of maternal and perinatal mortality and morbidity. Although it affects only 2–8% of pregnancies, over 70,000 women and 500,000 children die from it each year. The exact etiology of preeclampsia is unclear; it is often referred to as a disease of theories and hypotheses. This paper reviews the most significant hypotheses and studies that aim to explain the etiology of preeclampsia. This may help identify new research paths and concepts that could bring us closer to understanding the exact etiology of preeclampsia. The complexity of pathogenetic relationships and mechanisms, heterogeneous clinical presentations, and the development of underlying changes early in pregnancy when patients are clinically asymptomatic and appear healthy are among the main reasons for difficulty identifying the exact causes of preeclampsia. Furthermore, preeclampsia is specific to human pregnancy; there is no ideal animal study model whose results could be fully extrapolated to humans. A more holistic approach that combines all the information, hypotheses, and pathogenetic relationships may offer hope for understanding why preeclampsia occurs and how to prevent and treat it effectively. A better understanding of the precise etiology of the condition holds promise for developing new options for the early diagnosis, effective prevention, and modern causal treatment of preeclampsia. This would reduce the risk of severe complications in affected patients and could have enormous implications for clinical practice.

## 1. Introduction

Over the past few decades, enormous research efforts have been devoted to deciphering the etiology of preeclampsia. Numerous groundbreaking studies have been conducted to better understand this condition, which is specific to human pregnancy. However, the etiology of preeclampsia remains unclear, often being referred to as a disease of theories and hypotheses [1,2].

Preeclampsia is a multisystem disorder whose development during pregnancy is associated with the abnormal adaptation of a woman’s cardiovascular system and impaired placental development [1,3]. Although preeclampsia affects only 2–8% of pregnancies [2], it remains a leading cause of maternal and perinatal mortality and morbidity, and 76,000 women and 500,000 children die from it each year [1]. Maternal, placental, and fetal factors, as well as genetic, immunological, and metabolic predispositions, underlie its development. It is generally believed that the development of preeclampsia requires the presence of the placenta, and the characteristic clinical features (hypertension with maternal organ dysfunction) result from involvement of the blood vessels and vascular endothelium, the largest organ, leading to multi-organ damage [2].

A modified two-stage model of preeclampsia development assumes that stress on the placental syncytiotrophoblast plays a key role in its development [1,3,4]. Changes and abnormalities occur in the first trimester of pregnancy, when the pregnancy appears to be progressing normally and the patient shows no clinical signs of disease. In the first stage, there is abnormal and insufficient trophoblast invasion, as well as abnormal spiral artery remodeling [3,4]. However, the resulting clinical symptoms, known as “maternal syndrome,” are characterized by an excess of antiangiogenic factors [5,6,7,8] and appear in the second half of pregnancy, specifically in the second and third trimesters [3]. It seems that hypoxia, oxidative stress, abnormal natural killer cells at the maternal–fetal interface, and genetic and environmental factors are also important causative factors for preeclampsia [5,9]. Overproduction of reactive oxygen species and depletion of antioxidant systems can lead to oxidative stress, cell damage, lipid peroxidation, DNA oxidation, and, consequently, cell dysfunction [9]. A better understanding of the precise etiology of the condition holds promise for developing new options for the early diagnosis, effective prevention, and modern causal treatment of preeclampsia. This would reduce the risk of severe complications in affected patients and could have enormous implications for clinical practice.

## 2. Preeclampsia

### 2.1. Definition and Diagnostic Criteria, Clinical Presentation

Preeclampsia is a multisystem disorder with widespread vascular dysfunction [10]. The definition of preeclampsia, based on two typical symptoms—hypertension, defined as systolic blood pressure ≥ 140 mmHg and/or diastolic blood pressure ≥ 90 mmHg measured twice at least 4 h apart, and daily proteinuria ≥ 0.3 g/day [2,10]—has been expanded to include other criteria.

According to the classifications of the International Society for the Study of Hypertension in Pregnancy (ISSHP) and the American College of Obstetricians and Gynecologists (ACOG), preeclampsia is characterized by the first appearance of hypertension after 20 weeks of pregnancy (and up to 6 weeks postpartum) in a previously normotensive patient and the presence of at least one indicator of internal organ damage in the pregnant woman, such as daily proteinuria ≥ 0.3 g/day, acute kidney injury defined as creatinine level ≥ 1.1 mg/dL, thrombocytopenia (PLT < 100,000/μL), elevated transaminase activity, sometimes with pathognomonic pain in the right upper quadrant of the abdomen as a pathognomonic symptom of HELLP syndrome (hemolysis, elevated liver enzymes, and low platelet count), hemolysis, DIC (disseminated intravascular coagulation), neurological symptoms or new cerebral/visual disturbances in the form of headaches, visual disturbances, dark spots before the eyes, tonic–clonic seizures (eclampsia), stroke or pulmonary edema, cerebral hemorrhage or even maternal death [2,10,11,12,13]. Preeclampsia may occur with or without symptoms of placental dysfunction in the form of fetal growth restriction (FGR) and/or symptoms of fetal distress [2,10,11,12,13].

Preeclampsia is a leading cause of maternal and neonatal morbidity and mortality. It also leads to hospitalization in intensive care units, cesarean sections, preterm placental abruption, intrauterine fetal growth restriction, preterm birth, and neonatal complications resulting from prematurity [10,14].

An increased risk of preeclampsia has been observed in nulliparous women; women with preeclampsia in a previous pregnancy or with a family history of preeclampsia; women treated for hypertension, diabetes, obesity, kidney disease, metabolic syndrome, or autoimmune diseases; and women after infertility treatment [10].

Currently, delivery is the only known causal treatment for preeclampsia. However, this is not always beneficial for the fetus, especially if it occurs far from the estimated due date. Preterm childbirth is associated with a higher risk of respiratory distress syndrome, intraventricular hemorrhage, sepsis, and bronchopulmonary dysplasia in infants [15]. In pregnancies complicated by early-onset preeclampsia, the risk of intrauterine fetal death is up to seven times higher than in healthy, normotensive pregnancies [16].

Preventing complications resulting from prematurity includes prenatal administration of glucocorticosteroids and magnesium sulfate, depending on gestational age. There is growing evidence that preeclampsia also has long-term negative effects on offspring. Newborns from pregnancies complicated by preeclampsia, especially those with fetal growth restriction, are at an increased risk for neurodevelopmental disorders, insulin resistance, diabetes, cardiovascular complications, and hypertension [14,15].

### 2.2. Subtypes of Preeclampsia

Taking into account the gestational age at the time of the first symptoms, there are two forms of clinically recognized preeclampsia: early-onset preeclampsia (symptoms appear before 34 weeks of pregnancy) and late-onset preeclampsia (when symptoms appear after 34 weeks of pregnancy) [11,17]. The division of preeclampsia into early and late onset is not only based on the gestational age at which clinically overt preeclampsia occurs but also on certain differences between these two subtypes of preeclampsia. Early preeclampsia affects only 5 to 20 percent of patients with preeclampsia-complicated pregnancies, and it is the result of both shallow invasion of the extravillous trophoblast with reduced transformation of the spiral arteries and changes in maternal blood flow entering the intervillous space with higher than normal blood flow velocity from abnormally transformed spiral arteries. These changes are also observed in pregnancies complicated by FGR, which occurs in only about 20% of pregnancies complicated by preeclampsia. Most cases (80–95% of all cases of preeclampsia worldwide) belong to the late subtype of preeclampsia, which is usually a purely maternal syndrome with little effect on the baby [2]. Both subtypes of preeclampsia (early and late onset) show no differences in terms of hypertension and proteinuria, characteristics that directly define preeclampsia [18]. Early-onset preeclampsia, compared to late-onset preeclampsia, is associated with a more severe course, greater dynamics of blood pressure increase, more severe proteinuria and organ complications—generally a worse prognosis for the pregnant woman [19,20,21,22] and a significantly more frequent co-occurrence of fetal growth restriction with accompanying symptoms of chronic hypoxia, which can even lead to intrauterine fetal death [23,24,25]. The earlier in pregnancy the symptoms of preeclampsia occur, the greater the risk of complications for the mother, fetus, or newborn. However, it should be emphasized that the aforementioned maternal risks may occur more frequently in patients with preeclampsia at term (80% of patients) [22].

## 3. Shallow Trophoblast Invasion in the Etiopathogenesis of Preeclampsia

### 3.1. Stage 1: Abnormal Placental Development and Trophoblast Invasion

The mechanism of abnormal placentation is controversial, but animal models have shown that uteroplacental ischemia leads to hypertension and multi-organ failure, which are observed in maternal preeclampsia [5,8]. Placental hypoperfusion leads to the production and release of vasoactive factors, resulting in the activation and damage of the vascular endothelium [2]. Excessively shallow trophoblast invasion, along with impaired transformation of the uterine spiral arteries and abnormalities in placental villi development, leads to placental dysfunction. This results in increased oxidative stress parameters and systemic vascular endothelial dysfunction. These changes manifest as clinical symptoms in later stages of the disease.

Normal placental development is characterized by the migration of highly invasive trophoblast cells beyond the chorion into the uterine mucosa. These cells then penetrate to a depth of approximately one-third of the uterine muscle. This process results in the remodeling of spiral arterioles into a low-resistance vascular system. This transformation ensures normal fetal growth and progression of the pregnancy [26].

An adequate oxygen concentration gradient between the placenta and maternal arteries is critical for this process to function properly. It has been suggested that the abnormal trophoblast invasion and placental hypoxia observed in preeclampsia result from an imbalance of oxygen and disorders of the methionine–homocysteine cycle [26]. The branched vascular network is crucial for the development of the placenta. This network depends on factors such as vascular endothelial growth factor (VEGF), placental growth factor (PlGF), angiopoietin-1 (Ang-1), angiopoietin-2 (Ang-2), soluble fms-like tyrosine kinase 1 (sFlt-1), and soluble endoglin (sEng) to regulate blood vessel growth [27]. The syncytiotrophoblast is the main site of production of sFlt-1, PlGF, and sEng, and it secretes these substances in large quantities into the maternal bloodstream [28,29]. During pregnancy, these factors are released from the syncytiotrophoblast into the maternal circulation to adapt the cardiovascular system to the demands of pregnancy [27]. In preeclamptic patients, sFlt-1 and sEng placental production is enhanced [30]. Imbalances in these factors can lead to abnormal placental vascular development.

An increased secretion of antiangiogenic factors can lead to an antiangiogenic state in the mother, which contributes to pregnancy pathologies such as preeclampsia and fetal growth restriction. There are many theories regarding the causes of placental dysfunction, including oxidative stress, abnormal natural killer cells at the maternal–fetal interface, and genetic and environmental influences. Numerous studies confirm that an abnormal placenta releases soluble active factors into the maternal circulation, causing inflammation, vascular endothelial dysfunction, and systemic maternal disease [6,7,30,31].

During a normal pregnancy, cytotrophoblast cells migrate into the maternal uterine spiral arteries as the placenta implants. These cells form vascular sinuses that provide the fetus with adequate nutrition and oxygenation. This infiltration progresses deep into the spiral arteries, reaching the level of the myometrium. This results in the extensive remodeling of the maternal spiral arterioles into low-resistance, high-capacity, high-flow vessels [5,30,32,33].

In pregnancies complicated by preeclampsia, abnormal placentation occurs when the cytotrophoblast fails to transform from a proliferative epithelium to an invasive endothelium. This leads to incomplete remodeling of the spiral arteries [34]. Inadequate remodeling of the spiral arteries leads to maternal vasoconstriction and relative placental ischemia [35]. Narrow spiral arteries are susceptible to atherosclerotic changes, including the presence of lipid-laden macrophages in the vessel lumen, fibrinous necrosis of the arterial wall, and mononuclear perivascular infiltrates [36]. These changes further compromise placental perfusion.

These changes are detected during a non-invasive Doppler ultrasound examination of the uterine arteries. They are characterized by a significant impairment of diastolic flow with a distinct notch in the waveform, as opposed to the normal, strong, undisturbed flow in both systolic and diastolic uterine arteries typically observed during physiological pregnancy. These abnormalities are observed in the preclinical phase and may be used to stratify patients into risk groups, indicating the initiation of preeclampsia prophylaxis [37,38].

Trophoblast abnormalities alone are believed to lead to shallow invasion and abnormal spiral artery transformation, resulting in placental ischemia and preeclampsia [34]. In pregnancies complicated by preeclampsia, atherogenic changes have also been observed in the radial arteries supplying the decidual arteries [39,40]. Decidual vasculopathy with acute atherosclerotic changes, medial hyperplasia, and perivascular lymphocytes is observed in placental insufficiency. This condition is associated with poorer clinical prognoses, higher diastolic blood pressure, impaired renal function, and an increased risk of fetal death [41].

In the third trimester of a normal pregnancy, the vessels of the decidua are characterized by a flat endothelium and a loss of vascular smooth muscle. However, in preeclampsia, secondary atherosclerotic changes occur in the decidual vessels: edematous and loosely arranged vascular endothelium, hypertrophy of the intima and media, and a lack of modification of vascular smooth muscle. This is a characteristic feature of decidual vasculopathy [39]. The question remains whether decidual vasculopathy is the cause of stage 1 in the pathogenesis of preeclampsia or whether these changes result from systemic damage to the maternal vascular endothelium secondary to the observed changes [39].

Another factor that appears to lead to preeclampsia is the abnormal transformation of the endometrium and decidua [42]. Absent or abnormal decidualization in vitro, as well as genetic defects, have been observed in patients with preeclampsia, which indicates a genetic influence [43]. It has been suggested that cells from the decidua may play an important role in reducing trophoblast invasion.

Hypoxia is also thought to play a key role in the pathogenesis of preeclampsia [44]. The low-oxygen environment during the early stages of gestational sac implantation favors trophoblast proliferation and blastocyst implantation in the uterus. The connection between the trophoblast and the spiral arteries creates spaces called intervillous sinuses, which allow for the inflow of maternal blood. This leads to an increase in oxygen tension and generates oxidative stress. Thus, it enables the differentiation of the proliferative trophoblast into an invasive phenotype that disrupts and remodels the spiral arteries [45].

Hypoxia-inducible factors (HIFs) are highly expressed in the proliferating trophoblast tissues and placentas of women with preeclampsia. Using a mouse model, researchers observed that overexpressing HIF-1α disrupts the trophoblast’s transformation from a proliferative state to an invasive state. This leads to hypertension, proteinuria, and restricted fetal growth [46]. Conversely, inhibiting HIF-1α with the estradiol metabolite 2-methoxyestradiol blocks the production of sFlt-1, a potent antiangiogenic factor responsible for the clinical symptoms of preeclampsia [47]. Studies have confirmed an association between lower placental perfusion fraction and fetal growth restriction, Doppler flow abnormalities in maternal and fetal vessels, lower neonatal weight, and higher sFlt-1 levels [48]. Additionally, the significance of oxidative stress in the trophoblast invasion process for the development of normal pregnancies or those complicated by preeclampsia has been highlighted.

Proper oxygen delivery through maternal blood flow after prior oxygen restriction is necessary for normal placentation. However, periodic changes in hypoxia and reoxygenation may lead to poor or overly shallow invasion of the spiral arteries and oxidative stress [30].

Another factor that occurs in preeclampsia is an imbalance between enzymes that generate reactive oxygen species (ROS) and antioxidants. This imbalance favors the enzymes that generate ROS, which contributes to the inhibition of trophoblast invasiveness. This occurs through increased expression and activity of ROS-generating enzymes and the inhibition of the Wnt/β-catenin pathway. It also promotes antiangiogenic factors, such as sFlt-1 [30,47]. Furthermore, decreased expression of superoxide dismutase and glutathione peroxidase, along with impaired antioxidant mechanisms, has been observed in women experiencing preeclampsia during pregnancy compared to healthy pregnant women [30].

It is believed that the increase in O2 after the 11th week of pregnancy stimulates trophoblast differentiation [49] and promotes their transition from a proliferative to an invasive phenotype, leading to a complete remodeling of the spiral arteries. The establishment of uteroplacental circulation allows for adequate delivery of oxygen and nutrients to the placenta and developing fetus [50].

Free radicals, superoxide, hydroxyl radicals, and hydrogen peroxide are the main forms of reactive oxygen species (ROS) [50]. They are produced from various sources in cells, including NADPH oxidases, xanthine oxidase, cytochrome P450 enzymes, the endoplasmic reticulum, and mitochondria [51,52,53,54]. Mitochondria are considered the main source of ROS in both physiological and pathological conditions.

It has been observed that inhibition of trophoblast invasion leads to uteroplacental ischemia. It seems that hypoxia and its adverse effects on the hypoxia-sensitive mitochondria of the uteroplacental unit lead to the overproduction of reactive oxygen species and thus to oxidative stress [50]. At low concentrations, ROS act as signaling molecules in the regulation of various cellular processes. However, the accumulation/excess of ROS causes damage to cellular proteins, lipid peroxidation, and DNA damage, and consequently cell damage [50]. Mitochondrial dysfunction promotes trophoblast apoptosis with reduction in its invasiveness, impairs spiral artery remodeling, and increases production of proinflammatory cytokines, such as TNF-α, IL-1β, and IL-6, and inhibits the release of placental hormones (estrogens, chorionic gonadotropin, and placental lactogen). In response to hypoxia, mitochondrial ROS induce hypoxia-inducible factors HIF-1α and HIF-2α, which stimulate the production of soluble tyrosine kinase (sFlt-1) in trophoblasts [55,56].

These active substances disrupt the normal functioning of the placenta. HIF-responsive miR-210s reduce the expression of iron-sulfur cluster scaffolding in the electron transport chain, increasing mitochondrial ROS production, leading to an increase in vascular smooth muscle tension. These changes induced by hypoxia and mitochondrial ROS overproduction underlie the pathogenesis of preeclampsia and FGR [50].

Syncytiotrophoblast is the first cell line exposed to oxygen-rich maternal blood. At the same time, it is susceptible to ROS due to insufficient levels of antioxidant enzymes such as manganese superoxide dismutase [57]. Elevated levels of reactive oxygen species inactivate lipids, proteins, and nucleic acids, causing lipid peroxidation, oxidation of amino acid residues (especially cysteine residues), protein formation and cross-linking, and oxidative DNA damage [58], which alters cellular metabolism. Oxidative stress appears to be the main factor causing vascular endothelial dysfunction and excessive trophoblast apoptosis, causing ischemic–reperfusion injury with increased xanthine oxidase activity, which produces high levels of reactive oxygen species (ROS), and also leading to an increase in antiangiogenic factors such as soluble fms-like tyrosine kinase-1 and soluble endoglin (sEng), which bind and neutralize circulating proangiogenic vascular endothelial growth factor (VEGF) and transforming growth factor β1 (TGF-β1) [59,60,61,62,63]. The most important sources of ROS are mitochondria, the endoplasmic reticulum (ER), and NADPH oxidase [59].

Abnormal remodeling of spiral arteries, placental hypoperfusion, and vascular endothelial dysfunction occurring in preeclampsia lead to an imbalance of circulating proangiogenic and antiangiogenic factors, especially placental growth factor (PlGF) and sFlt-1, which leads to an increase in the sFlt-1/PlGF ratio [64,65,66]. Increased sFlt-1 and sEng and decreased PlGF lead to the development of the maternal syndrome of preeclampsia [64,65,66,67,68]. In normal pregnancy, low oxygen levels are observed in the first trimester, products of chronic hypoxia, and ROS increase VEGF expression mainly through HIF-1 and maintain PlGF at low oxygen levels, and then, as normal pregnancy progresses, increasing oxygen levels result in lower VEGF and increase PlGF [69,70]. In contrast, premature hemoperfusion and hyperoxia in early pregnancy can lead to decreased VEGF and a premature increase in PlGF, which is likely to underlie weaker villous vascular development, superficial trophoblast invasion, abnormal placental development, and pregnancy complications [69,70,71,72]. It is widely believed that preeclampsia is caused by vascular endothelial dysfunction triggered by active factors in the mother’s blood released from an ischemic/hypoxic placenta [73]. Periods of ischemia/reperfusion, which are a consequence/result of abnormal trophoblast invasion, lead to hypoxia, stress, and oxidative damage. In addition, impaired release of nitric oxide, a recognized vasodilator from the vascular endothelium, and an imbalance in favor of ROS appear to play an important role in the pathogenesis of preeclampsia [74,75,76].

Increased reactive oxygen species may result from mitochondrial stress. Zsengellér et al. [77] demonstrated reduced activity of the mitochondrial electron transport chain (ETC) and cytochrome C oxidase in syncytiotrophoblast cells of placentas from preeclamptic pregnancies, which correlated with increased sFLT1 expression in the placenta. Additionally, damage caused by sequential ischemia and reflow leads to endoplasmic reticulum (ER) stress in the decidua and placentas from pregnancies complicated by preeclampsia or fetal growth restriction, resulting in apoptosis of the decidua and cytotrophoblast cells, as well as reduced transcription of placental growth factor, a key proangiogenic factor essential for normal pregnancy development [78,79,80,81].

In rodents, the induction of heme oxygenase (HO-1) has been shown to have beneficial effects, including a reduction in blood pressure and an increase in the ratio of vascular endothelial growth factor (VEGF) to soluble fms-like tyrosine kinase 1 in the placenta [82]. Physiologically, the trophoblast coats the outer wall of the decidual capillaries and the interendometrial branches of the spiral arteries, forming the outer sheath of these vessels. Trophoblast cells infiltrate the capillary walls from the outside in, forming loose clusters within them. During the next phase of cytotrophoblast invasion, the endothelium and most of the musculoelastic fibers are lost. The endothelial cells of the spiral arteries are partially replaced by extravillous trophoblast cells within the blood vessels.

Remodeling of the spiral arteries occurs. During a normal pregnancy, the diameter of their lumen increases four- to six-fold, and they become insensitive to vasopressors due to nerve fiber degradation. These changes affect the spiral arteries in both the decidual and myometrial portions. The vascular endothelium layer is restored, and wide uteroplacental arteries form. This ensures low-pressure, low-resistance blood flow, which promotes proper perfusion and oxygenation of the intervillous space [83,84,85].

In preeclampsia, pseudovasculogenesis fails, causing cytotrophoblast cells to fail to adopt an invasive endothelial phenotype. Consequently, the invasion of spiral arteries is incomplete and limited to the decidua, failing to reach the intrauterine myometrium. These arteries remain small-diameter, high-resistance vessels, which leads to placental ischemia [30].

Abnormal spiral arteries are narrower but retain their reactivity. Placental perfusion and the maternal–placental–fetal exchange surface are reduced, and the volume of placental villi shrinks. Blood flow is high-resistance, with variable velocity and turbulence during inflow, which further damages the villi surface [83,84,85]. Oxidative stress increases, leading to the release of proinflammatory cytokines and vasoactive factors, which in turn activate and dysfunction the vascular endothelium [29]. Both early- and late-onset preeclampsia result from stress on the placental syncytiotrophoblast [3].

### 3.2. Stage 2 Involves an Imbalance in Circulating Angiogenic Factors and Underlies the Development of Maternal Syndrome

An imbalance in circulating angiogenic factors is responsible for the maternal symptoms of preeclampsia. Currently, elevated levels of soluble fms-like tyrosine kinase-1 and soluble endoglin, along with significantly reduced levels of placental growth factor, shift the balance in favor of antiangiogenic factors and induce microangiopathy in target organs such as the kidneys, liver, and brain, giving rise to preeclampsia [30,67,68].

These substances are primarily produced in the syncytiotrophoblast and secreted into the maternal circulation [29,85,86,87,88,89]. Overly shallow placentation and abnormal vascular remodeling, along with the associated ischemic processes and placental damage, are responsible for the increased secretion of antiangiogenic factors into the maternal circulation [86,87,88,89]. An imbalance of these factors leads to vascular changes and microangiopathy in vital organs, especially those with fenestrated endothelium, such as the brain, kidneys, or liver.

sFlt-1 is a soluble protein that exerts anti-angiogenic effects by binding to and inhibiting the biological activity of pro-angiogenic proteins such as VEGF and PlGF [30]. VEGF is important for maintaining endothelial cell function. PlGF, on the other hand, plays an essential role in angiogenesis and selectively binds to VEGFR1/sFLT-1, but not to VEGFR2 [90,91]. The administration of exogenous sFlt1 to rodents resulted in hypertension, proteinuria, and glomerular endotheliosis—hallmarks of preeclampsia. In contrast, reducing sFlt1 levels or antagonizing sFlt1 in experimental animal models of preeclampsia attenuated clinical symptoms [92,93] or led to their spontaneous resolution when sFlt-1 levels were reduced by half or more by treating underlying placental conditions such as fetal hydrops or by removing diseased placentas in multiple pregnancies [90,91,94,95].

Soluble endoglin (sEng), another antiangiogenic protein that has been extensively studied in preeclampsia, is an endogenous inhibitor of TGF-β1 (transforming growth factor β1) [96]. Elevated levels of soluble endoglin (sEng), an endogenous inhibitor of transforming growth factor β1 (TGF-β1), have been detected in the serum of women with preeclamptic pregnancies as early as two months before the onset of clinical symptoms. These levels correlate with disease severity, leading to fetal growth retardation, thrombocytopenia, and, in combination with sFlt-1, cerebral edema [68,97,98].

### 3.3. Cytokines and Changes in Immune Cells

Preeclampsia is well-known to be a proinflammatory condition; however, the responsible mechanism has not yet been fully elucidated. It seems that syncytiotrophoblast microvesicles and exosomes, which are rich in sFlt1 and endoglin, may initiate an inflammatory response [99,100]. A normal pregnancy is characterized by a shift in the T cell phenotype toward Th2 versus Th1 [101,102]. However, in pregnancies complicated by preeclampsia, an abnormal shift toward the Th1 phenotype is observed, which leads to insufficient trophoblast invasion [103]. Additionally, a preeclampsia-like syndrome can be induced in healthy pregnant rats by transferring CD4+ cells derived from RUPP models.

Studies on the peripheral blood mononuclear cells of women with preeclampsia have shown reduced secretion of IL-10. This may lead to impaired T lymphocyte differentiation because IL-10 is a cytokine that induces T lymphocyte differentiation into the Th2 phenotype (T helper type 2). IL-10 has properties that neutralize proinflammatory cytokines, AT1-AA (autoantibodies against the angiotensin II receptor 1), and placental ROS (reactive oxygen species) and ET-1 (endothelin-1) [101,102,103,104,105,106,107].

Preeclampsia has also been shown to be associated with elevated complement levels and C3 genetic mutations [30,108,109]. Animal studies have demonstrated that inhibiting complement component activity restores spiral artery capacitance and reduces sFlt1 production in this patient group [30,108,109]. The highest level of abnormal complement activity is observed in HELLP syndrome, which is similar to atypical hemolytic uremic syndrome, which is associated with uncontrolled complement activation [30].

### 3.4. Renin–Angiotensin–Aldosterone System

Increased sensitivity to angiotensin II has been reported both in clinically overt preeclampsia and in the preeclamptic period, despite reduced levels of circulating renin and angiotensin II compared with normal pregnancy [109,110,111,112,113,114].

Circulating anti-angiotensin II type 1 receptor (AT1) autoantibodies in women with preeclampsia may be a potential mechanism that increases sensitivity to angiotensin II [109,110,111,112,113,114]. These autoantibodies induce vasoconstriction via endothelin-1 (ET-1) activation, necrosis and apoptosis of umbilical vein endothelial cells, reduced trophoblast invasion, and increased reactive oxygen species (ROS) production, which stimulates tissue factors and leads to hypercoagulability [109,110,111,112,113,114].

Additionally, an increase in the number of CD19+CD5+ cells, as well as anti-angiotensin II type 1 receptor antibody activity (AT1-AA), has been observed in the serum of patients with preeclampsia. This suggests that B lymphocytes play a role in the development of preeclampsia. Anti-AT1-AA antibodies, produced by the CD19+CD5+ subpopulation in response to placental ischemia and systemic inflammation, appear to contribute to hypertension and the production of antiangiogenic factors that characterize the maternal syndrome. Antibodies against the angiotensin type 1 receptor, produced in response to placental ischemia and inflammation, stimulate production of antiangiogenic factors, such as sFlt-1 and sEng, in the placenta [30,113,114,115,116]. In animal models, elevated levels of circulating sFlt1 are sufficient to induce sensitivity to angiotensin II by interfering with the normal production of nitric oxide by the vascular endothelium.

### 3.5. Homocysteine

Reduced blood homocysteine levels are observed in pregnant women with normal pregnancies. Conversely, elevated homocysteine levels are associated with implantation disorders, embryogenesis abnormalities, neural tube defects, miscarriage, fetal death, premature placental abruption, hypertension, and fetal growth restriction (FGR) [3,30,115,116]. Furthermore, hyperhomocysteinemia leads to endothelial cell dysfunction, vascular wall damage, increased fibrosis, impaired blood flow, increased platelet activation, thrombosis, atherosclerotic changes, and abnormal placental function [115,116].

### 3.6. Nitric Oxide and ADMA

Nitric oxide (NO) is a key factor in regulating placental blood flow. It has potent vasodilatory effects and inhibits platelet aggregation and vascular smooth muscle proliferation. NO also reduces the release of free oxygen radicals and lowers vascular tone [115,116]. NO plays an active role in intravascular cytotrophoblast invasion and placental development due to its unique angiogenic properties [115,116].

Asymmetric dimethylarginine (ADMA) is an endogenous inhibitor of nitric oxide synthase (NOS) that has been associated with impaired endothelial function and uterine artery flow abnormalities observed in preeclampsia [67,115,116]. Elevated homocysteine (Hcy) levels in women with preeclampsia lead to increased ADMA levels due to Hcy’s inhibitory effect on ADMA metabolism. Nitric oxide (NO) also increases the proangiogenic activity of vascular endothelial growth factor and placental growth factor while decreasing the levels of soluble fms-like tyrosine kinase 1 in hypoxic human trophoblast cells [67,115,116]. In preeclampsia, reduced nitric oxide production in the fetoplacental unit leads to placental vasoconstriction, impaired placental perfusion, increased maternal blood pressure, and increased peripheral resistance [30,115,116]. Increased apoptosis and aponecrosis (a form of incomplete apoptosis) have also been reported in patients with preeclampsia. The continuous increase in villous turnover leads to intense proliferation and turnover of trophoblast cells, leading to increased flow of placental material into the maternal circulation, the presence of degenerative changes in the syncytiotrophoblast, and higher levels of fetal DNA in the maternal circulation. The maternal immune system is thought to play a significant role in the development of preeclampsia. One cause of abnormal trophoblast invasion in early pregnancy is believed to be an altered immune response of the pregnant woman, characterized by abnormal tolerance of the maternal immune system, comparable to the rejection reaction of an allogeneic transplant.

Sack et al. and Borzychowski and Luppi described the excessive activation of neutrophils and monocytes, as well as the increased spontaneous production of proinflammatory cytokines, such as IL-1β, IL-6, and IL-8, in patients with preeclamptic pregnancies [117,118,119]. Furthermore, an increased tendency toward a proinflammatory response in CD4+ and CD8+ T lymphocytes, natural killer (NK) cells, and dendritic cells due to dysregulation of Toll-like receptors. This response was similar to that observed in non-pregnant women but different from the immunosuppressive and anti-inflammatory response characteristic of healthy pregnant women [26,101,117,118,120]. Natural killer (NK) cells play an important role in regulating cellular interactions during physiological changes and promoting placental development and spiral artery transformation [117,118,120]. Decidual NK cells secrete fewer invasion-promoting factors in women with abnormal uterine artery flow examination results, which may explain the shallow invasion observed in pregnancies complicated by preeclampsia.

Genetics is another factor that contributes to the development of preeclampsia. Variations in the incidence of preeclampsia have been observed based on race, geography, and socioeconomic status. Women with first-degree relatives with preeclampsia have a five-fold increased risk, while those with second-degree relatives have a two-fold increased risk, and there is a two-fold increased risk in patients who themselves were born to pregnancies complicated by preeclampsia [26]. Abnormal trophoblast invasion, which occurs early in pregnancy in patients with certain genetic or immunological predispositions, appears to lead to increased vascular resistance in the uteroplacental circulation and altered resistance in the uterine arteries [26].

Persistent subperfusion leads to placental hypoxia, which induces local oxidative stress and increases apoptosis and necrosis of trophoblast villi. This, in turn, has fetal implications and may lead to the development of fetal growth restriction (FGR) or the release of vasoactive factors into the maternal circulation, leading to the next stage of preeclampsia, with a systemic inflammatory response and vascular endothelial dysfunction, which should be considered an interdependent, interactive process preceding the onset of clinically apparent preeclampsia symptoms [118].

The mechanisms responsible for abnormal placental development in preeclampsia (PE) remain incompletely understood. However, several factors have been suggested to play a role, including impaired maternal immune recognition, increased HIF-1α and HIF-2α, increased TGF-β3, altered soluble VEGF/PIGF receptor ratios, low levels of PIGF, and altered levels of angiogenic factors [26,30,31].

## 4. A Hypothesis of the Preeclampsia Etiology Takes into Account Maternal Susceptibility, Development of the Villous Trophoblast, and of the Extravillous Trophoblast

The hypothesis concerning the etiology of preeclampsia presented by B. Huppertz suggests that the placenta, the development of villous and extravillous trophoblasts, and the factors released from it should be considered in relation to the mother and her genetic susceptibility [2,119].

Normal development of the villous trophoblast releases apoptotic syncytial nodes that do not cause an inflammatory response in the mother, while a disturbed villous trophoblast leads to the release of necrotic and aponecrotic particles that can lead to systemic damage or activation of the mother’s vascular system [2,119,121]. Abnormal invasion of the extravillous trophoblast may be relevant to the development of preeclampsia, especially in pregnant women who are highly susceptible. Taking these three variables into account, various options for the development of preeclampsia have been proposed, taking into account different clinical forms of the condition, such as early preeclampsia, late preeclampsia, preeclampsia with appropriate for gestational age fetal growth, or preeclampsia complicated by FGR [2].

**Figure 1:**
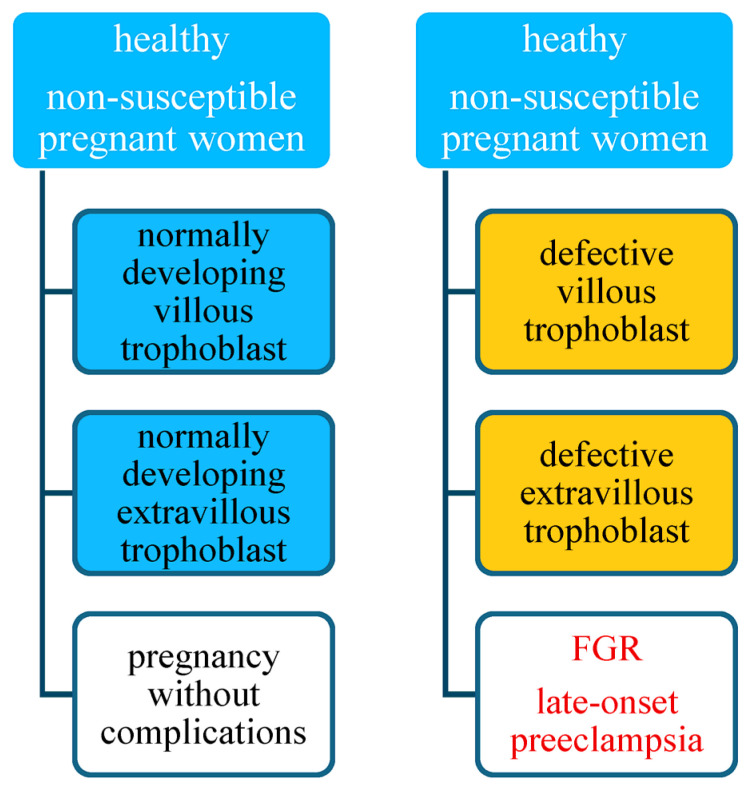

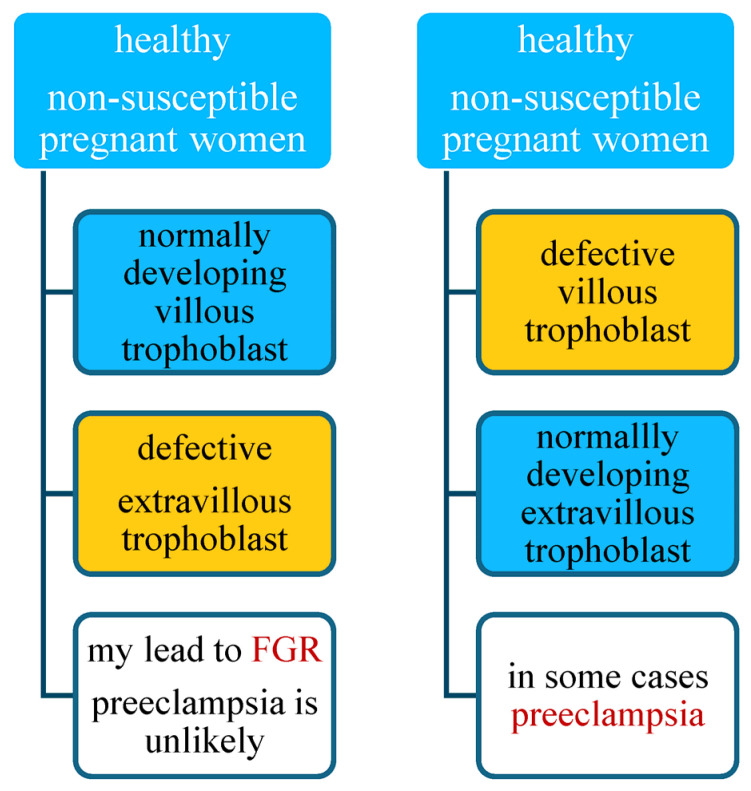
Risk of preeclampsia in non-susceptible pregnant women.

Pregnancy in a healthy woman with normal development of villous and extravillous trophoblast should proceed without complications and result in the birth of a healthy newborn. In susceptible women with normal development of the villous and extravillous trophoblast, it may lead to the development of preeclampsia, but FGR is unlikely [2,118]. [Data presented in Figure 1].

**Figure 2:**
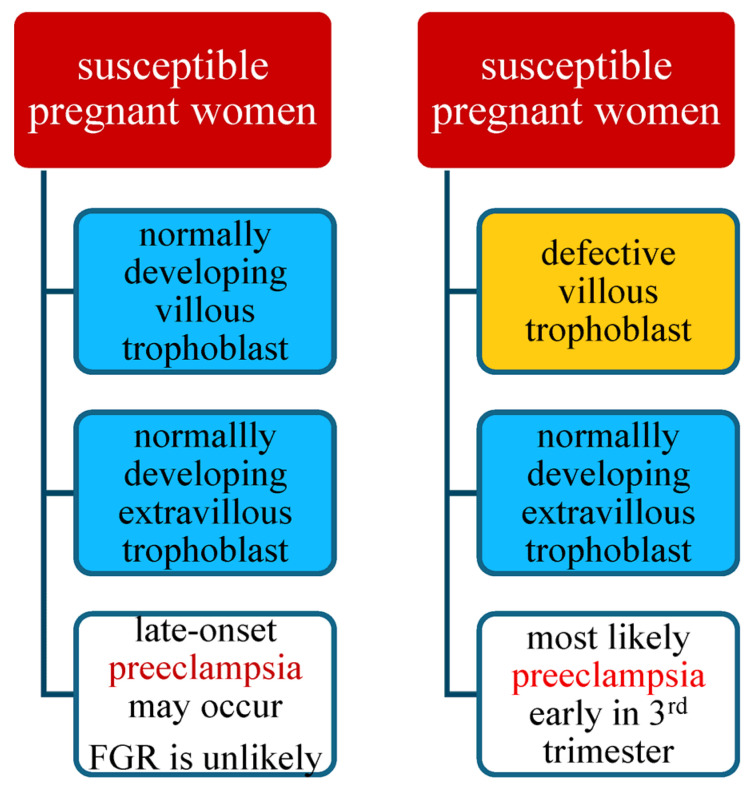

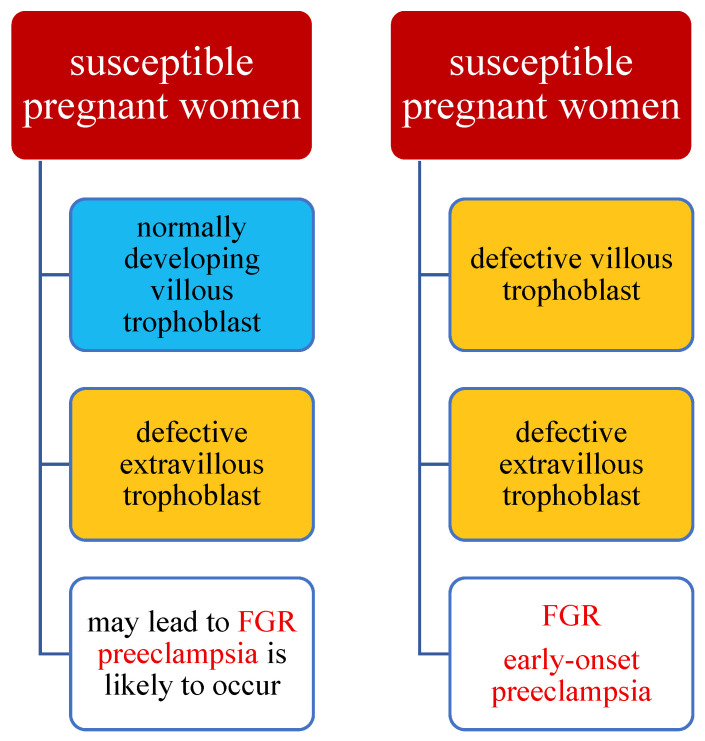
Risk of preeclampsia in susceptible pregnant women.

In the case of a susceptible mother with normal development of the villous and extravillous trophoblasts, late-onset preeclampsia may occur, most often without signs of FGR, especially when accompanied by a larger placental mass, e.g., in diabetes, multiple pregnancy, or macrosomic fetus. The development of late-onset preeclampsia depends on the degree of susceptibility of the pregnant woman, the overload of her defense and cleansing mechanisms, and the degree of damage to her vascular system. In this scenario, early-onset preeclampsia may also occur [2,118]. [Data presented in Figure 2].

In the case of a healthy pregnant woman with normal development of the extravillous trophoblast but defective development of the villous trophoblast, the pregnancy should result in the birth of a healthy infant with normal fetal growth but in some cases may lead to the development of preeclampsia.

Abnormal development of the trophoblast, in addition to releasing apoptotic syncytial nodes into the maternal blood [2,121,122], leads to the release of subcellular, necrotic, and aponecrotic fragments, proteins, and DNA, which activate the maternal endothelium and may ultimately lead to damage to the maternal vascular system, inflammatory response, and clinical symptoms of preeclampsia [2,123]. Normal development of the extravillous trophoblast ensures appropriate placental perfusion and fetal nutrition, thus ensuring proper fetal growth.

In the case of a healthy/non-susceptible pregnant woman with a normally developing villous trophoblast but a malformed extravillous trophoblast, the pregnancy may lead to fetal growth restriction, while the development of preeclampsia is unlikely. In this case, the normal release of apoptotic particles from the villous trophoblast is accompanied by insufficient invasion of the extravillous trophoblast into all structures, i.e., uterine arteries and veins, lymphatic vessels, as well as uterine glands, and abnormal transformation of spiral arteries [124]. The normal funnel-shaped widening of the spiral arteries at the end of the vessels reduces the maternal blood inflow velocity into the placenta to 10 cm/s [85,123]. This low inflow velocity does not damage the very delicate structures of the villous tree and allows blood to be distributed through the narrow spaces between the villi without detaching the villi anchored in the basal plate.

In contrast, blood flow from the non-dilated spiral arteries to the placenta is 10–20 times higher than in a normal pregnancy and has a velocity of approximately 1–2 m/s [85,123,124]. The increased speed of maternal blood flowing into the placenta damages the delicate villi of the placenta, causing fibrin deposits to form, the villi to detach from the basement membrane, and changes in the structure of the villi. This can lead not only to fetal growth retardation but even to spontaneous miscarriage at a very early stage of pregnancy. This option is also associated with increased peripheral resistance in the small vessels of the placental villi, changes in perfusion, poorer supply of oxygen and nutrients to the fetus, and fetal malnutrition.

In the case of a susceptible woman with normal development of the extravillous trophoblast and abnormal development of the villous trophoblast, a healthy baby is born without fetal growth retardation, but this will most likely lead to the development of preeclampsia early in the third trimester of pregnancy [2]. However, in the case of a susceptible pregnant woman with a normally developing villous trophoblast but a defective extravillous trophoblast, the pregnancy may lead to fetal growth restriction, and preeclampsia is also likely to occur [2].

Pregnancy in a healthy, non-susceptible woman with defective development of both the villous and extravillous trophoblast will result in the birth of a growth-restricted baby, and the mother will suffer from late-onset preeclampsia. The worst-case scenario concerns a susceptible patient with a defective placenta with abnormal development of both the villous and extravillous trophoblast. Such pregnancies have the highest burden because of the risk of intrauterine fetal growth restriction in the course of early-onset preeclampsia [125]. Figure 1 and Figure 2.

## 5. Clinical Implications

Research on imbalances in maternal angiogenic factors and their impact on vascular function has found application in methods for detecting and stratifying the risk of early-onset preeclampsia. Testing for levels of sFlt-1, sEng, and PlGF has found particular application in predicting the onset of preeclampsia and assessing its severity. Elevated sFlt-1 levels and decreased PlGF levels, as well as an increase in the sFlt-1/PlGF ratio, can be used to predict the development of PE later in pregnancy or the risk of severe complications of preeclampsia [65,66]. The sFlt-1/PlGF ratio is widely recognized as a better marker of preeclampsia than any of these substances individually and is useful in predicting the onset of preeclampsia, assessing prognosis, and differential diagnosis in conditions similar to preeclampsia. sFlt-1/PlGF values below 38 allow PE and its occurrence within the next 4 weeks to be ruled out in over 95% of cases. Results above 85 or 110 at a gestational age above 34 weeks indicate a high probability of PE [30,126,127].

Extremely high results, i.e., above 655 or even 201, are alarming and suggest active intervention, as 70% of patients will require delivery within the next 48 h due to the high risk of complications [126,127].

The results of studies on animal models using recombinant human PlGF and siRNA are also promising, as this may be an effective therapy, especially in early preeclampsia, allowing the risk of premature delivery in this group of patients to be avoided or reduced [30,126,127].

A more reliable test than dipstick tests for urine screening, which is available more quickly than daily urine output assessment, is the assessment of the albumin-to-creatinine ratio (ACR) in a single urine sample. An ACR result of <30 mg/mmol virtually rules out significant proteinuria, while a value of ≥30 mg/mmol requires verification in a 24 h urine collection. In clinical practice, a test using Congo red in urine is used, which is characterized by a high positive predictive value in identifying preeclampsia and high repeatability. It is a simple test that can be used at the patient’s bedside to confirm the diagnosis of preeclampsia in women with suspected disease.

The prediction of preeclampsia also involves the examination of blood flow in the uterine and placental arteries assessed from 20 weeks of gestation and the determination of intrauterine fetal growth and development until the end of pregnancy (early initial ultrasound examination and subsequent regular check-ups). These studies could contribute to the implementation of prophylaxis of preeclampsia with acetylsalicylic acid at a dose of 100–150 mg per day from 12–16 to 34–36 weeks of gestation, taken orally in the evening.

Unfortunately, despite intensive research, there is still no satisfactory treatment that can halt the progression of preeclampsia, except for immediate delivery. This is not beneficial for the fetus, especially in cases of early-onset preeclampsia, as it often carries the unavoidable consequences of prematurity. Therefore, safe and effective methods that prolong pregnancy without risk to the mother would be beneficial [128]. The first experimental therapies using apheresis in the treatment of preeclampsia, proposed by Winkler and Wang, were based on extracorporeal heparin-mediated extracorporeal low-density-lipoprotein precipitation (HELP) apheresis as a possible therapeutic approach in preeclampsia.

The basis for implementing this experimental therapy, lipid apheresis [129], was data from the literature regarding the role of angiogenic imbalance and lipoprotein metabolism in the etiopathogenesis of preeclampsia. Normotensive pregnancy is characterized by a lipid profile with an atherogenic phenotype, marked by an increase in both triglycerides and cholesterol in all lipoprotein fractions—VLDL, LDL, and HDL [129,130,131,132,133,134]. These changes are more pronounced in pregnancies complicated by preeclampsia [130,131,132,133]. High triglyceride levels are associated with a four-fold higher incidence of preeclampsia [134,135], and their content in low-density lipoproteins (LDLs) [129,135,136] correlates positively with diastolic blood pressure and proteinuria. Furthermore, it has been suggested that not only antiangiogenic imbalance with an excess of antiangiogenic factors, primarily sFlt-1 and sEng, but also changes in the lipid and lipoprotein profile and their metabolism are among the main factors causing placental perfusion disorders, as confirmed by the “acute atherosclerosis” changes observed in placental vessels [132]. It was also observed that lipoprotein levels positively correlated with disease severity, the risk of thrombotic processes, placental hypoxia, and maternal endothelial dysfunction, likely due to the susceptibility of small, dense LDL particles to oxidative modification [132]. Additionally, after LDL apheresis, a decrease in soluble tyrosine kinase 1 (sFlt1), an antiangiogenic factor that is crucial for the pathophysiology of maternal syndrome, was observed [133]. Wang’s study also observed a reduction in maternal proinflammatory markers, blood coagulation parameters, and plasma viscosity without any apparent adverse effects in either the mother or the neonate following treatment with HELP-apheresis [132]. These authors used HELP—apheresis in nine patients with preeclampsia. This procedure allowed them to prolong pregnancy from 3 to 49 days—an average of 17.7 days. Good neonatal outcomes were also achieved. Unfortunately, one infant died from late-onset sepsis [132].

Similarly, Thadhani et al. conducted a study on the efficacy and safety of therapeutic plasma apheresis with a negatively charged dextran sulfate column to remove positively charged sFlt-1. The study demonstrated an increase in clearance and a decrease in blood sFlt-1 concentration by 7 to 28%, a reduction in proteinuria with a concomitant 44% reduction in the protein/creatinine ratio, and an extension of gestation by an average of 8 or 15 days in women undergoing single or multiple therapeutic apheresis compared with 3 days in patients with early-onset preeclampsia between 23 and 32 weeks of gestation not undergoing apheresis [137]. No serious adverse consequences of therapeutic apheresis for either the mother or the fetus were observed in this study [137].

The results of these clinical trials are encouraging, demonstrating that the symptoms of preeclampsia can be alleviated and pregnancy safely prolonged with plasmapheresis. However, these results should be interpreted with great caution, as they were usually non-randomized studies on small groups of patients, or rather, on individual patients, and the results were not always conclusive. Without a randomized approach, one cannot expect an equal number of patients in each group, and without a blinded approach, one cannot expect objective decision-making regarding the timing of delivery, often based on the well-informed but subjective assessment of experienced obstetricians [137].

Furthermore, the study conducted by Haddad et al. was stopped for safety reasons after enrolling two patients, as both developed secondary, uncontrolled hypertension and blurred vision during treatment [138]. Although transient clinical improvement was observed with reduced proteinuria and reduced need for antihypertensive medication within the first 36 h following LDL apheresis, the sudden and uncontrolled increase in clinical symptoms quickly led to cesarean delivery in both patients with early and severe preeclampsia, and LDL apheresis did not reduce serum sFlt1 levels in these two patients. The first neonate, born at 26 weeks of gestation, died of sepsis on the 5th day of life, and the second, born at 27 weeks of gestation, is still alive and well. The main limitation of this study was the inclusion of only two patients.

Good cooperation between the cardiologist/hypertension specialist and the gynecologist–obstetrician, as well as a clear flow of information on both sides regarding abnormalities in the course of pregnancy, is crucial for pregnancy with hypertension or preeclampsia, especially preeclampsia with severe features.

According to the guidelines, echocardiography in pregnant women with hypertension is not mandatory but should be considered at the first visit to the cardiologist if a patient with hypertension has a new, previously non-existent, unexplained cardiac symptom at any stage of pregnancy [10,139,140,141].

Preeclampsia is a disease of the uterus and other arterial beds, with extensive vascular dysfunction. It is increasingly seen not only as a complication of pregnancy but also as a significant/critical risk factor for cardiovascular disease/conditions later in a woman’s life, including hypertension, ischemic heart disease, stroke, and heart failure (HF). These patients are at significantly increased risk of developing chronic hypertension within 10 years after delivery. Preeclampsia often also precedes other cardiovascular risk factors, such as hypertension, diabetes, and obesity, which contribute to an increased risk profile in these women as they age. The mechanisms linking preeclampsia to cardiovascular disease remain poorly defined [10,139,140,141].

This increased risk appears to be more than just a side effect of pregnancy. Rather, the same pathophysiological mechanisms underlying preeclampsia and cardiovascular disease are at play, including endothelial dysfunction, inflammation, and metabolic abnormalities. Women with severe or recurrent preeclampsia, or who have given birth prematurely, are at an even greater risk. Some evidence suggests that they are two times more likely to develop cardiovascular disease than women with uncomplicated pregnancies. The American Heart Association now includes preeclampsia in its cardiovascular risk assessment guidelines, emphasizing the importance of monitoring blood pressure and cholesterol levels and making lifestyle modifications as early strategies for preventing cardiovascular disease in women with a history of this condition.

Children born from pregnancies complicated by preeclampsia, apart from the risk of short-term complications resulting mainly from prematurity, are exposed to an increased risk of neurodevelopmental disorders, insulin resistance, diabetes, cardiovascular disease, and hypertension in adulthood [14,15,16,50].

## 6. Conclusions

Despite numerous studies, the etiology of preeclampsia has not yet been fully elucidated. A better understanding of the condition’s precise etiology holds promise for the development of new options for early diagnosis, prevention, and modern causal treatment of preeclampsia. Research on maternal angiogenic factor imbalances and their effect on vascular function has led to the development of methods for detecting and assessing the risk of early-onset preeclampsia. Levels of sFlt-1, sEng, and PlGF have proven to be particularly useful in predicting the occurrence and severity of preeclampsia [26,30,142].

Furthermore, clinical trials have demonstrated that preeclampsia symptoms can be alleviated and pregnancy can be safely prolonged through plasma apheresis, which removes antiangiogenic proteins [128,129,133,136,137,143]. Studies in animal models using recombinant human PlGF and siRNA have also shown promise as an effective therapy, particularly for early-onset preeclampsia. This therapy could help reduce the risk of severe complications and premature delivery in affected patients [26,30,144,145,146].

## Data Availability

No new data were created or analyzed in this study.
